# Quadratus lumborum block at the lateral supra-arcuate ligament for postoperative analgesia: a protocol for a systematic review and meta-analysis

**DOI:** 10.1080/07853890.2025.2525406

**Published:** 2025-06-30

**Authors:** Zhonghua Li, Ling Zhang, Qin Zhang, Liang Yu, Yu Shen, Keyu Fan, Mingxia Liu, Dongxu Wang, Ya Cao, Yuxuan Zhang, Lu Qian, Danru Wu, He Liu, Jiewei Xu

**Affiliations:** a Department of Anesthesiology & Clinical Research Center for Anesthesia and Perioperative Medicine & Key Laboratory of Anesthesia and Analgesia Application Technology, Huzhou Central Hospital, The Fifth School of Clinical Medicine of Zhejiang Chinese Medical University, Huzhou, China; b Huzhou Central Hospital, The Affiliated Central Hospital Huzhou University, Huzhou, China; c Affiliated Huzhou Hospital, Zhejiang University School of Medicine, Huzhou, China; d Huzhou Key Laboratory of Basic Research and Clinical Translation for Neuromodulation, Huzhou Central Hospital, The Fifth School of Clinical Medicine of Zhejiang Chinese Medical University, Huzhou, China; e MOE Key Lab for Neuroinformation, University of Electronic Science and Technology of China, Chengdu, China; f Department of General Surgery, Huzhou Central Hospital, The Fifth School of Clinical Medicine of Zhejiang Chinese Medical University, Huzhou, China

**Keywords:** Quadratus lumborum block, lateral supra-arcuate ligament, postoperative pain, analgesia, recovery

## Abstract

**Background:**

The quadratus lumborum block at the lateral supra-arcuate ligament (QLB-LSAL) is a recent development in the field of regional anaesthetic techniques, offering several advantages over the traditional quadratus lumborum block. However, most current studies on this topic are single-centre and small-sample studies, which may limit the evaluation of the efficacy and safety of this block method.

**Methods:**

This protocol adopts the Preferred Reporting Items for Systematic Reviews and Meta-Analyses (PRISMA) guidelines. This evaluation aims to assess the efficacy and safety of QLB-LSAL for postoperative analgesia. The primary outcome is the total consumption of opioid morphine equivalents within 24 h post-surgery. Secondary outcomes comprised numerical rating scale (NRS) pain scores within 24 h postoperatively, Quality of Recovery-15 (QoR-15) scores, patient satisfaction scores, opioid-related side effect incidence and block-related adverse events. This study will only include randomized clinical trials. We will conduct electronic database searches based on the established search strategy. The two review authors will independently screen the literature, extract data and use the Cochrane Risk of Bias 2 tool to assess the risk of bias. We will conduct a meta-analysis on the extracted data and assess the risk of bias for each study. We will also conduct trial sequential analysis, sensitivity analysis and subgroup analysis and assess the overall risk of publication bias. Finally, the GRADE guidelines will be used to assess the certainty of the evidence.

**Discussion:**

The latest systematic review methodology will be used to assess the efficacy and safety of QLB-LSAL for the treatment of postoperative pain. The findings are expected to provide valuable insights for clinicians to apply QLB-LSAL in the management of postoperative analgesia.

## 1. Introduction

Postoperative pain can have a significantly impede the patient’s recovery, potentially leading to adverse outcomes and hindering effective recuperation following surgery. Contributing factors may include tissue injury resulting from the surgical incision and pain sensitization due to secondary inflammatory response. Inadequate pain control during the acute postoperative period may also contribute to chronic pain development [1,2]. Patient-controlled intravenous analgesia using opioid medications remains the primary approach for postoperative pain management. However, opioid administration is associated with significant adverse reactions, including respiratory depression, skin pruritus and neurological complications characterized by drowsiness, hallucinations and myoclonus. Moreover, Opioid drugs can result in postoperative intestinal obstruction, which is not a direct mechanical blockage but a type of functional intestinal paralysis or opioid-induced bowel dysfunction (OIBD) [3].

Multimodal analgesia plays a crucial role in the concept of the enhanced recovery after surgery (ERAS) [4,5]. In summary, combining non-opioid analgesics with regional anaesthetic techniques enhances pain management and reduces opioid dosage, thereby minimizing the occurrence of associated adverse effects. Regional block techniques can be categorized into three main types: neuraxial (subarachnoid or epidural), peripheral nerve block and fascial plane block. Currently, the most commonly used regional blocks for post-thoracic and abdominal surgical pain management include epidural analgesia, paravertebral nerve block, transverse abdominal plane block, erector spinae block, quadratus lumborum block (QLB) and local infiltration analgesia. However, these analgesic methods often have limitations such as increased incidence of complications, incomplete pain relief efficacy, challenging surgical procedures implementation and significant interindividual variability [6–9].

The quadratus lumborum block at the lateral supra-arcuate ligament (QLB-LSAL) is a novel nerve block technique involving local anaesthetic injection above the lateral arcuate ligament at the anterior aspect of the quadratus lumborum muscle [10]. This approach enables direct drug diffusion into the lower thoracic paravertebral endothoracic fascia space, minimizing individual anatomical variations that typically affect drug distribution. The technique facilitates more efficient drug spread into the paravertebral space, resulting in a broader blockade range, faster onset and reduced complications [10,11]. Clinical applications include unilateral abdominal surgery, unilateral retroperitoneal organ procedures and total abdominal surgery with bilateral blockade [9,11–16].

In conclusion, the management of postoperative analgesia using QLB-LSAL technology presents numerous potential benefits. However, it is important to note that the majority of recent research in this field has been conducted at a single centre and with a limited sample size. This limitation may hinder the comprehensive assessment of the safety and effectiveness of this block method. Consequently, conducting a systematic review and meta-analysis of existing literature will be undertaken to strengthen the evidence base supporting the therapeutic applicability of this method.

### 1.1. Objective

1. Data extraction: Establish research eligibility criteria, conduct literature screening and extract data according to the predetermined literature retrieval strategy following PRISMA guidelines.
2. Data analysis: Perform meta-analysis using Revman5.4 software on the extracted data and assess the risk of bias for each study using the Cochrane Risk of Bias Tool version 2.0.
3. Data synthesis: Conduct trial sequential analysis, sensitivity analysis and subgroup analysis on the extracted data to evaluate the overall risk of publication bias. Assess the quality of evidence using the GRADE guidelines.

The aim is to evaluate the effectiveness and safety of the QLB-LSAL technique in postoperative pain management compared to other analgesic methods.

## 2. Method

### 2.1. Eligibility criteria

#### 2.1.1. Study design

This plan will be prepared according to the Preferred Reporting Items for Systematic Review and Meta-Analysis Protocols (PRISMA-P) statement [17] (checklist in the supplement A). This evaluation has been registered with the International Prospective Register of Systematic Reviews (PROSPERO), CRD42024595383 [18]. We will follow the systematic review methods established by the Cochrane Collaboration [19] and utilize the Grading of Recommendations Assessment, Development and Evaluation (GRADE) [20] framework for reporting purposes, following PRISMA statement [17].

#### 2.1.2. Type of studies

The study will exclusively encompass randomized controlled clinical trials that employ QLB-LSAL technology for the purpose of postoperative analgesia. Only studies published in the English language will be included. In case any unpublished trials are identified, we shall furnish the pertinent data and method descriptions through written means or by directly contacting the authors.

#### 2.1.3. Participants

We will include patients who meet the following criteria:

1. Age ≥18 years
2. Surgical intervention in the abdominal region
3. Requirement for postoperative pain management

#### 2.1.4. Types of interventions

Experimental group: The effectiveness of postoperative analgesia using QLB-LSAL technology is independent of the specific formulation, dosage, or concentration of local anaesthetics employed, and it is not influenced by the surgeon’s proficiency.

Control group: All alternative analgesic techniques, including intravenous patient-controlled analgesia with opioids, other regional nerve blocks (such as quadratus lumborum block, transversus abdominis plane block, thoracic epidural anaesthesia) and combinations of these methods.

### 2.2. Information sources and search strategy

#### 2.2.1. Electronic searches

The following electronic biomedical databases will be explored by us:

- PubMed
- Embase
- Web of science
- Cochrane Library
- Wanfang Data,
- China National Knowledge Infrastructure (CNKI)

The retrieval period is defined as the time span from the establishment of the database until 20 December 2024. The search will be conducted within six months after the manuscript is submitted for system evaluation.

#### 2.2.2. Searching other sources

We will conduct a thorough screening to identify any trials that may have been overlooked during the database searches. Additionally, we will conduct a comprehensive search for ongoing clinical trials and unpublished studies.

- ClinicalTrials.gov
- The World Health Organization International Clinical Trials Registry Platform (ICTRP)
- The ISRCTN registry (www.isrctn.com)
- Chinese Clinical Trial Registry (ChiCTR) (www.chictr.org.cn/)
- Clinical Trials Information System (CTIS)

#### 2.2.3. Search strategy

The search strategy has been specifically designed for use with PubMed and will be adapted for other databases. The search strategy was developed with the assistance of information experts. The search strategy is presented in the supplement B.

### 2.3. Outcomes

#### 2.3.1. Primary outcome

Total consumption of intravenous morphine equivalents (IVME) within the first 24 h following surgery.

#### 2.3.2. Secondary outcomes

- Numerical rating scale (NRS) pain score, satisfaction score, time to first flatus, time to oral intake, time to ambulation and time to discharge within 24 h postoperatively;
- Opioid-related adverse effects, including postoperative nausea and vomiting (PONV), itching, hypotension, chills and respiratory depression (<10 breaths/min);
- The incidence of block-related adverse events, including diaphragmatic injury, haematoma, local anaesthetic systemic toxicity (LAST) and lower limb weakness.

### 2.4. Data collection and analysis

#### 2.4.1. Selection of studies

The title and abstract of each study are independently reviewed by two members of the research team. In case of any discrepancies, they will engage in consultation to reach a consensus or involve a third author for discussion in order to resolve the issue.

#### 2.4.2. Data extraction

The following data are extracted from the included trials (if available):

- Basic information: the authors’ names, title of the publication, date of publication and language used;
- The characteristics of trial participants encompass the patient population, inclusion and exclusion criteria, as well as the setting;
- Intervention (methods of anaesthesia, dosage and concentration of local anaesthetic, administration techniques, ultrasound-guided procedures);
- Results (as described above);
- Data on risk of bias (as specified below);
- Sponsorship (trial funding and potential conflicts of interest among trial authors);
- The available data from duplicate publications, supplementary documents, or multiple reports of the main study will be utilized to maximize the information yield.

#### 2.4.3. Risk of bias assessment

The two review authors will independently employ the Cochrane Risk of Bias Tool 2.0 (RoB2) [21] to evaluate the risk of bias in the selected studies for the randomized controlled trials. We will examine the following domains of bias risk:

- The presence of bias in the randomization process;
- Refraining from the established intervention introduces a potential bias;
- arising from missing outcome data;
- Bias in the measurement of outcomes;
- The presence of bias in the selective reporting of results.

The RoB2 flowchart facilitates the categorization of risk of bias in each domain into three levels: ‘low risk of bias’, ‘some concerns’ and ‘high risk of bias’. Subsequently, the overall risk of bias is assessed based on the evaluation of bias in all domains, which is also divided into three levels: ‘low risk of bias’, ‘some concerns’ and ‘high risk of bias’. The judgements regarding the risk of bias will be presented in the risk of bias table, while the supporting information will provide reasons for each judgement.

### 2.5. Data synthesis

#### 2.5.1. Effect measurements

For dichotomous outcomes, we will calculate the relative risk (RR) along with its corresponding 95% confidence interval (CI), *p* value and trial sequential analysis (TSA)-adjusted 95% CIs.

For continuous outcomes, we will compute the mean difference (MD) together with its corresponding 95% CI, *p* value and TSA-adjusted 95% CIs.

A meta-analysis will be conducted when there are at least two studies providing comparable effect measurement results and heterogeneity measurements indicate appropriateness of summary results. If the outcome data are unsuitable for meta-analysis, effect estimates will be summarized following Chapter 12 guidelines from the Cochrane Handbook for Systematic Reviews of Intervention [22].

#### 2.5.2. Missing data

In accordance with the Cochrane Handbook for Systematic Reviews of Interventions, we will minimize direct data imputation. If experimental data, methodology details, or result measurements are missing, we will first contact the original researchers to request the information. Missing data will be addressed according to the type of missingness. In cases of random missing data, we will analyse only available data. For non-random missing data, we will employ imputation techniques, such as calculating statistics based on worst-case scenarios or using mean substitution. Subsequently, we will conduct a sensitivity analysis to evaluate the impact of missing data replacement on result stability and robustness under specified conditions. If the original researchers cannot be reached, we will exclude their research data from this analysis. If missing data must be included, we will estimate standard deviations using methods outlined in Chapter 6 of the Cochrane Handbook for Systematic Reviews of Interventions [23]. Concurrently, we will perform sensitivity analyses to evaluate the potential impact of statistical assumption variations on study results.

#### 2.5.3. Assessment of heterogeneity

The primary approach for assessing heterogeneity between studies will involve visual examination of the forest plot, supplemented by the I^2^ test and Q statistic. In case heterogeneity is detected, an investigation will be conducted to verify the accuracy of data included in the original study, as well as evaluate the appropriateness of employed data extraction methods. If heterogeneity can potentially be attributed to factors such as race, treatment duration, or dosage, a subgroup analysis or meta-regression will be utilized. Furthermore, sensitivity analysis (see below) will help elucidate the sources of heterogeneity. Should statistical heterogeneity be deemed significant, a decision may be made to exclude meta-analysis and instead present descriptive data.

#### 2.5.4. Meta-analysis

We will use the software Revman 5.4 for conducting the meta-analysis [24], wherein appropriate statistical methods, effect models (random effects or fixed effects models), and effect indicators will be selected based on a comprehensive evaluation of the underlying assumptions. A single primary outcome will be determined, with a significance threshold set at *p* value of .05. The Type I error rate will not be adjusted for both primary and secondary outcomes.

#### 2.5.5. Trial sequential analysis

The risk of false positives or false negatives in the meta-analysis will be assessed using TSA [25], which corrects for the increased risk of Type I and Type II errors resulting from multiple data mergers and provides the required sample size for the meta-analysis. By establishing sequential monitoring boundaries, we ensure that the overall Type I error is limited to 5%. We have set alpha at 5% and beta at 20%. TSA will be applied to our main results in the meta-analysis.

#### 2.5.6. Sensitivity analysis

The robustness of each step and its impact on the merger results will be assessed through a sensitivity analysis, which is anticipated to be conducted. A one-at-a-time (OAT) approach will be employed for this purpose. Sequential exclusion of one article at a time using Revman 5.4 software will precede the meta-analysis performed on the remaining articles (n − 1). Evaluation of individual studies’ influence on the combined results aims to determine if specific studies significantly affect the original meta-analysis findings. In case heterogeneity between studies becomes insignificant after excluding a particular piece of literature, further analysis will explore clinical and methodological differences between that literature and others to identify factors influencing heterogeneity.

#### 2.5.7. Subgroup analysis

The subgroup analysis of the primary outcomes will be conducted in accordance with the methodology outlined below.

- Low bias risk trials vs. high bias risk trials;
- Based on postoperative analgesia type (including quadratus lumborum block, transversus abdominis plane block, thoracic epidural analgesia, or other analgesia strategies, etc.);
- Based on surgical type (including liver surgery, kidney surgery, rectal surgery, obstetric surgery, etc.);
- Based on ropivacaine dosage (higher or lower than the median dose used in the trials included for each outcome);
- Based on the choice of ropivacaine concentration;
- Evaluated age groups (≥65 years old adults vs. <65 years old adults);
- Trials with profit bias vs. trials without profit bias.

#### 2.5.8. Confidence in cumulative evidence

The GRADE methodology will be utilised in order to assess the certainty of the evidence [20]. According to the GRADE evidence profile, the certainty of evidence may be reduced if risk of bias, imprecision (small sample populations), inconsistency (heterogeneity), indirectness (including surgical types), or publication bias is found to exist.

## 3. Discussion

In general, postoperative pain can be divided into two categories: somatic pain, which originates from the skin incision and visceral pain, which originates from the internal organs, muscles and bones. Among these, visceral pain has been described as lacking in sharpness and characteristically diffused, with an inability to localize the source [26]. The pathological mechanisms of visceral pain are complex and involve surgical manipulation, peritoneal inflammation, visceral ischaemia and visceral hypersensitivity due to sensitization of peripheral and central pathways [27]. Visceral pain can trigger autonomic reflexes, potentially triggering a cascade of nausea and vomiting, as well as cardiovascular effects and potentially increasing negative emotions in the patient [28]. Additionally, early postoperative visceral pain is an independent risk factor for the development of chronic, unexplained pain [29]. Therefore, seeking solutions to visceral pain is a proper way to improve the quality of postoperative analgesic management.

QLB is frequently combined with general anaesthesia for abdominal or hip surgeries, potentially reducing intraoperative anaesthetic dosage, mitigating postoperative pain and facilitating patient rehabilitation [30–33]. The mechanism of action involves local anaesthetic diffusion from the quadratus lumborum interfascial plane through the diaphragm’s lower medial and lateral arcuate ligaments to the thoracic paravertebral space [34–37]. However, the presence of these ligamentous barriers limits anaesthetic spread, predominantly confining the drug solution to the fascial plane beneath the arcuate ligament. Consequently, this anatomical constraint may result in prolonged onset time, increased local anaesthetic requirements, variable efficacy and elevated risk of local anaesthetic toxicity. Furthermore, surgical interventions on retroperitoneal organs can compromise the thoracolumbar fascia’s structural integrity, significantly impacting the block’s effectiveness [38,39].These factors limit its application to some extent.

QLB-LSAL represents an advanced regional blockade technique derived from traditional QLB, which mitigates individual anatomical variations in muscle, fascia and neural pathways, thereby enhancing anaesthetic precision [10]. There are multiple implementation methods for QLB-LSAL technique, with paravertebral short-axis and long-axis scanning techniques being the most widely used in clinical practice [10,40]. The short-axis technique involves positioning the ultrasound probe vertically to the spinal midline, 3–4 cm lateral to the T_12_–L_1_ transverse section, to perform a paramedian scan of the paraspinal interval. The target puncture area is the triangular space formed by the intersecting courses of the diaphragm and quadratus lumborum muscles [10]. The long-axis technique involves scanning an ultrasound probe 6–8 cm along the quadratus lumborum muscle’s side attachment at the T_12_ rib, then moving towards the spine’s midline. During this movement, identify the T_12_ rib and L_1_ transverse process tip [40]. Between these points, the deep surface of the quadratus lumborum muscle can be observed moving with respiration. The space between the quadratus lumborum muscle and diaphragm serves as the target area for puncture [40]. Moreover, a novel blocking technique targets the drug diffusion area at the junction of the diaphragmatic arch muscle and quadratus lumborum, particularly suitable for patients with low pleural positions [41]. The ultrasound scanning positioning method closely resembles the paravertebral midline longitudinal axis technique, with emphasis on identifying the anterior layer of the thoracolumbar fascia extending continuously from the diaphragm muscle’s deep surface towards the caudal region. The optimal puncture target is the gap between the quadratus lumborum and the anterior layer of the thoracolumbar fascia situated below the lateral arcuate ligament [41]. Given that most relevant randomized controlled clinical studies have utilized the paramedian midline longitudinal axis blockade technique, we will adopt this approach in our observational cohort.

Several studies have been conducted to compare the advantages and disadvantages of QLB-LSAL with conventional regional block techniques in terms of efficacy and safety for postoperative analgesia. However, to date, no systematic review has ever evaluated the efficacy and safety of QLB-LSAL for postoperative analgesia. The strengths of this systematic evaluation include the development of the study protocol in accordance with PRISMA guidelines, which were developed in line with the systematic review methodology of the Cochrane Collaboration and GRADE. Furthermore, the evaluation was conducted in accordance with PRISMA reporting guidelines. Other strengths include the predefined patient-relevant outcomes and the assessment of confidence in pre-specified subgroup analyses. Additionally, the evaluation employed the TSA method to assess the risk of random error.

Nevertheless, there are potential constraints to this systematic evaluation. We anticipate clinical heterogeneity among the included studies, particularly with respect to setting and population, which may impede the feasibility of conducting meta-analyses. To address this issue, we will assess clinical heterogeneity using I^2^ analyses with predefined subgroup analyses based on patient age, type of surgery and clinical setting.

## 4. Conclusions

This systematic evaluation will delineate the degree of certainty in the evidence pertaining to the efficacy and safety of QLB-LSAL for postoperative analgesia. The findings will inform the identification of new research questions and provide background data for the design of future trials, thereby establishing a theoretical basis for future involvement in multimodal analgesia management.

## Supplementary Material

Supplementary_Material.docx

## Data Availability

Data sharing is not applicable to this article as no data were created or analysed in this study.

## References

[1] Pogatzki-Zahn EM, Segelcke D, Schug SA. Postoperative pain-from mechanisms to treatment. Pain Rep. 2017;2(2):e588. doi:10.1097/PR9.0000000000000588. 29392204 PMC5770176

[2] Richebé P, Capdevila X, Rivat C. Persistent postsurgical pain: pathophysiology and preventative pharmacologic considerations. Anesthesiology. 2018;129(3):590–607. doi:10.1097/ALN.0000000000002238. 29738328

[3] Zhang Z, Li C, Xu L, et al. Effect of opioid-free anesthesia on postoperative nausea and vomiting after gynecological surgery: a systematic review and meta-analysis. Front Pharmacol. 2023;14:1330250. doi:10.3389/fphar.2023.1330250. 38239201 PMC10794765

[4] Viscusi ER, Epelde F, Roca Ruiz LJ, et al. Present and future of pharmacological management for acute moderate-to-severe postoperative, traumatic, or musculoskeletal pain in Europe: a narrative review. Pain Ther. 2024;13(6):1351–1376. doi:10.1007/s40122-024-00645-y. 39305453 PMC11543979

[5] Chassery C, Atthar V, Marty P, et al. Opioid-free versus opioid-sparing anaesthesia in ambulatory total hip arthroplasty: a randomised controlled trial. Br J Anaesth. 2024;132(2):352–358. doi:10.1016/j.bja.2023.10.031. 38044236

[6] Huang X, Wang J, Zhang J, et al. Ultrasound-guided erector spinae plane block improves analgesia after laparoscopic hepatectomy: a randomised controlled trial. Br J Anaesth. 2022;129(3):445–453. doi:10.1016/j.bja.2022.05.013. 35803754

[7] Grape S, Kirkham KR, Akiki L, et al. Transversus abdominis plane block versus local anesthetic wound infiltration for optimal analgesia after laparoscopic cholecystectomy: a systematic review and meta-analysis with trial sequential analysis. J Clin Anesth. 2021;75:110450. doi:10.1016/j.jclinane.2021.110450. 34243030

[8] El-Boghdadly K, Pawa A. The erector spinae plane block: plane and simple. Anaesthesia. 2017;72(4):434–438. doi:10.1111/anae.13830. 28188611

[9] Shi R, Shao P, Hu J, et al. Anterior quadratus lumborum block at lateral supra-arcuate ligament vs lateral quadratus lumborum block for postoperative analgesia after laparoscopic colorectal surgery: a randomized controlled trial. J Am Coll Surg. 2024;238(2):197–205. doi:10.1097/XCS.0000000000000897. 37861219

[10] Li H, Ma D, Liu Y, et al. A transverse approach for ultrasound-guided anterior quadratus lumborum block at the lateral supra-arcuate ligament. Anaesthesia. 2020;75(10):1400–1401. doi:10.1111/anae.15058. 32578192

[11] Guo M, Lei B, Li H, et al. Anterior quadratus lumborum block at the lateral supra-arcuate ligament versus transmuscular quadratus lumborum block for analgesia after elective cesarean section: a randomized controlled trial. J Clin Med. 2022;11(13):3827. doi: 10.3390/jcm11133827. PMC9267333 35807110

[12] Li H, Shi R, Shi D, et al. Anterior quadratus lumborum block at the lateral supra-arcuate ligament versus transmuscular quadratus lumborum block for postoperative analgesia in patients undergoing laparoscopic nephrectomy: a randomized controlled trial. J Clin Anesth. 2021;75:110561. doi:10.1016/j.jclinane.2021.110561. 34798706

[13] Mao Y, Zhao W, Hao M, et al. Ultrasound-guided quadratus lumborum block at the lateral supra-arcuate ligament versus subcostal transversus abdominis plane block for postoperative analgesia following open hepatectomy: a randomized controlled trial. J Pain Res. 2023;16:1429–1440. doi:10.2147/JPR.S404810. 37138955 PMC10150756

[14] Gu B, Zhou H, Lian Y, et al. Ultrasound-guided anterior quadratus lumborum block at lateral supra-arcuate ligament vs thoracic epidural analgesia after open liver surgery: a randomized, controlled, noninferiority trial. J Am Coll Surg. 2022;235(6):871–878. doi:10.1097/XCS.0000000000000354. 36102582

[15] Liu S, Cao L, Zhang Y, et al. Application of ultrasound-guided anterior quadratus lumborum block approach at the lateral supra-arcuate ligament in elderly patients undergoing colorectal cancer surgery. Am J Cancer Res. 2024;14(9):4248–4264. doi:10.62347/BOZK1723. 39417167 PMC11477835

[16] Lin L, Yu Y, Ke P, et al. Ultrasound-guided bilateral anterior quadratus lumborum block at the lateral supra-arcuate ligament in patients undergoing laparoscopic radical gastrectomy: a randomised controlled study. J Perioper Pract. 2025;35(3):77–87. doi:10.1177/17504589241242341. 39991806

[17] Shamseer L, Moher D, Clarke M, et al. Preferred reporting items for systematic review and meta-analysis protocols (PRISMA-P) 2015: elaboration and explanation. BMJ. 2015;350:g7647. doi:10.1136/bmj.g7647. 25555855

[18] Zhonghua Li, Liang Yu, He Liu. Anterior quadratus lumborum block at the lateral supra-arcuate ligament for postoperative analgesia : a systematic review and meta-analysis. PROSPERO 2024. https://www.crd.york.ac.uk/PROSPERO/view/CRD42024595383 10.1080/07853890.2025.2525406 PMC12210404 40586695

[19] Higgins JPT, Thomas J, Chandler J, et al. Cochrane Handbook for Systematic Reviews of Interventions. 2nd Edition. Chichester (UK): John Wiley & Sons, 2019.

[20] Granholm A, Alhazzani W, Møller MH. Use of the GRADE approach in systematic reviews and guidelines. Br J Anaesth. 2019;123(5):554–559. doi:10.1016/j.bja.2019.08.015. 31558313

[21] Sterne JAC, Savović J, Page MJ, et al. RoB 2: a revised tool for assessing risk of bias in randomised trials. BMJ. 2019;366:l4898. doi:10.1136/bmj.l4898. 31462531

[22] McKenzie JE, Brennan SE. Chapter 12: synthesizing and presenting findings using other methods. In: Higgins JPT, Thomas J, Chandler J (editors). Cochrane Handbook for Systematic Reviews of Interventions. 2nd Edition. Chichester (UK): John Wiley & Sons, 2019.

[23] Higgins JPT, Li T, Deeks JJ, et al. Chapter 6: choosing effect measures and computing estimates of effect. In: Higgins JPT, Thomas J, Chandler J (editors). *Cochrane Handbook for Systematic Reviews of Interventions*. 2nd Edition. Chichester (UK): John Wiley & Sons, 2019.

[24] Harrogate SR, Barnes JD, Gupta S, et al. Protocol for a systematic review and meta-analysis of tobacco-cessation interventions delivered perioperatively. BMJ Open*.* 2023;13(9):e067722. 10.1136/bmjopen-2022-067722 PMC10510911 37714672

[25] Temberg JL, Pisljagic S, Steensbaek MT, et al. Mixing short- and long-acting local anaesthetics in peripheral nerve blocks: Protocol for a systematic review and meta-analysis. Acta Anaesthesiol Scand*.* Mar 2024;68(3):417–422. 10.1111/aas.14352 37947347

[26] Boezaart AP, Smith CR, Chembrovich S, et al. Visceral versus somatic pain: an educational review of anatomy and clinical implications. Reg Anesth Pain Med. 2021;46(7):629–636. doi:10.1136/rapm-2020-102084. 34145074

[27] Johnson AC, Greenwood-Van Meerveld B. The pharmacology of visceral pain. Adv Pharmacol. 2016;75:273–301. doi:10.1016/bs.apha.2015.11.002. 26920016

[28] Yang GW, Cheng H, Song XY, et al. Effect of oxycodone-based multimodal analgesia on visceral pain after major laparoscopic gastrointestinal surgery: a randomised, double-blind, controlled trial. Drug Des Devel Ther. 2024;18:1799–1810. doi:10.2147/DDDT.S464518. PMC11141770 38828025

[29] Blichfeldt-Eckhardt MR, Ording H, Andersen C, et al. Early visceral pain predicts chronic pain after laparoscopic cholecystectomy. Pain. 2014;155(11):2400–2407. doi:10.1016/j.pain.2014.09.019. 25250720

[30] Corso RM, Piraccini E, Sorbello M, et al. Ultrasound-guided transmuscular quadratus lumborum block for perioperative analgesia in open nephrectomy. Minerva Anestesiol. 2017;83(12):1334–1335. doi:10.23736/S0375-9393.17.12167-X. 28679202

[31] Ince I, Hamadnalla H, Hassan M, et al. Ultrasound-guided quadratus lumborum plane block for congenital hip dislocation surgery: dermatomes and osteotomes. J Clin Anesth. 2019;54:140. doi:10.1016/j.jclinane.2018.10.044. 30508809

[32] Hansen C, Dam M, Nielsen MV, et al. Transmuscular quadratus lumborum block for total laparoscopic hysterectomy: a double-blind, randomized, placebo-controlled trial. Reg Anesth Pain Med. 2021;46(1):25–30. doi:10.1136/rapm-2020-101931. 33082286

[33] Andersen CHS, Laier GH, Nielsen MV, et al. Transmuscular quadratus lumborum block for percutaneous nephrolithotomy: study protocol for a dose-finding trial. Acta Anaesthesiol Scand. 2020;64(8):1224–1228. doi:10.1111/aas.13605. 32297653

[34] Adhikary SD, El-Boghdadly K, Nasralah Z, et al. A radiologic and anatomic assessment of injectate spread following transmuscular quadratus lumborum block in cadavers. Anaesthesia. 2017;72(1):73–79. doi:10.1111/anae.13647. 27730633

[35] Balocco AL, López AM, Kesteloot C, et al. Quadratus lumborum block: an imaging study of three approaches. Reg Anesth Pain Med. 2021;46(1):35–40. doi:10.1136/rapm-2020-101554. 33159007

[36] Dam M, Moriggl B, Hansen CK, et al. The pathway of injectate spread with the transmuscular quadratus lumborum block: a cadaver study. Anesth Analg. 2017;125(1):303–312. doi:10.1213/ANE.0000000000001922. 28277325

[37] Tamura T, Yokota S, Ito S, et al. Local anesthetic spread into the paravertebral space with two types of quadratus lumborum blocks: a crossover volunteer study. J Anesth. 2019;33(1):26–32. doi:10.1007/s00540-018-2578-5. 30413879

[38] Li H, Shi R, Wang Y. Use of transmuscular quadratus lumborum block for postoperative analgesia after laparoscopic nephrectomy. Reg Anesth Pain Med. 2021;46(12):1118–1119. doi:10.1136/rapm-2021-102478. 33632779

[39] Li H, Shi R, Wang Y. Transincisional ultrasound-guided quadratus lumborum block in open renal surgeries. Pain Phys. 2021;24(1):E127–E128. 33400446

[40] Shi R, Li H, Wang Y. Dermatomal coverage of single-injection ultrasound-guided parasagittal approach to anterior quadratus lumborum block at the lateral supra-arcuate ligament. J Anesth. 2021;35(2):307–310. doi:10.1007/s00540-021-02903-1. 33550463

[41] Li H, Shi R, Wang Y. A modified approach below the lateral arcuate ligament to facilitate the subcostal anterior quadratus lumborum block. J Pain Res. 2021;14:961–967. doi:10.2147/JPR.S306696. 33880061 PMC8053522

