# Supplementary material for: Quadratus lumborum block at the lateral supra-arcuate ligament for postoperative analgesia: a protocol for a systematic review and meta-analysis
Source: Ann Med. 2025 Jun 30;57(1):2525406. doi: 10.1080/07853890.2025.2525406 (PMC12210404; doi:10.1080/07853890.2025.2525406)
Supplement: Supplementary_Material.docx [file IANN_A_2525406_SM3339.docx]

**SUPPLEMENT**

**for**

**Quadratus lumborum block at the lateral supra-arcuate ligament for postoperative analgesia：A protocol for a systematic review and meta-analysis**

**SUPPLEMENT A**

**PRISMA-P (Preferred Reporting Items for Systematic review and Meta-Analysis Protocols) 2015 checklist: recommended items to address in a systematic review protocol***

| **Section and topic** | **Item No** | | | **Checklist item** |  |
| --- | --- | --- | --- | --- | --- |
| **ADMINISTRATIVE INFORMATION** | | | | |  |
| Title: |  | | |  |  |
| Identification | 1a | | | Identify the report as a protocol of a systematic review - page 1 |  |
| Update | 1b | | | If the protocol is for an update of a previous systematic review, identify as such - NA |  |
| Registration | 2 | | | If registered, provide the name of the registry (such as PROSPERO) and registration number – pages 7 |  |
| Authors: |  | | |  |  |
| Contact | 3a | | | Provide name, institutional affiliation, e-mail address of all protocol authors; provide physical mailing address of corresponding author – page 1-3 |  |
| Contributions | 3b | | | Describe contributions of protocol authors and identify the guarantor of the review  – page 19 |  |
| Amendments | 4 | | | If the protocol represents an amendment of a previously completed or published protocol, identify as such and list changes; otherwise, state plan for documenting important protocol amendments – page 7 |  |
| Support: |  | | |  |  |
| Sources | 5a | | | Indicate sources of financial or other support for the review – page 19 |  |
| Sponsor | 5b | | | Provide name for the review funder and/or sponsor – page 19 |  |
| Role of sponsor or funder | 5c | | | Describe roles of funder(s), sponsor(s), and/or institution(s), if any, in developing the protocol – page 19 |  |
| **INTRODUCTION** |  | | |  |  |
| Rationale | 6 | | | Describe the rationale for the review in the context of what is already known – page 5 and 6 |  |
| Objectives | 7 | | | Provide an explicit statement of the question(s) the review will address with reference to participants, interventions, comparators, and outcomes (PICO) – pages 7 |  |
| **METHODS** |  | | |  |  |
| Eligibility criteria | 8 | | | Specify the study characteristics (such as PICO, study design, setting, time frame) and report characteristics (such as years considered, language, publication status) to be used as criteria for eligibility for the review – pages 7 and 8 |  |
| Information sources | 9 | | | Describe all intended information sources (such as electronic databases, contact with study authors, trial registers or other grey literature sources) with planned dates of coverage – pages 8 and 9 |  |
| Search strategy | 10 | | | Present draft of search strategy to be used for at least one electronic database, including planned limits, such that it could be repeated – Supplement 1 and 2 |  |
| Study records: |  | | |  |  |
| Data management | 11a | | | Describe the mechanism(s) that will be used to manage records and data throughout the review – page 10 and 11 |  |
| Selection process | 11b | | | State the process that will be used for selecting studies (such as two independent reviewers) through each phase of the review (that is, screening, eligibility and inclusion in meta-analysis) – page 10and 11 |  |
| Data collection process | 11c | | | Describe planned method of extracting data from reports (such as piloting forms, done independently, in duplicate), any processes for obtaining and confirming data  from investigators – page 10 and 11 |  |
| Data items | | | 12 | List and define all variables for which data will be sought (such as PICO items,  funding sources), any pre-planned data assumptions and simplifications – pages 10 and 11 | |
| Outcomes and prioritization | | | 13 | List and define all outcomes for which data will be sought, including prioritization of main and additional outcomes, with rationale – pages 9 and 10 | |
| Risk of bias in individual studies | | | 14 | Describe anticipated methods for assessing risk of bias of individual studies, including whether this will be done at the outcome or study level, or both; state how this information will be used in data synthesis – page 11 | |
| Data synthesis | | | 15a | Describe criteria under which study data will be quantitatively synthesised – pages 11 - 12 | |
|  | | | 15b | If data are appropriate for quantitative synthesis, describe planned summary measures, methods of handling data and methods of combining data from studies, including any planned exploration of consistency (such as I^2^, Kendall’s τ) – pages 12 - 13 | |
|  | | | 15c | Describe any proposed additional analyses (such as sensitivity or subgroup analyses, meta-regression) – page 13 and 14 | |
|  | | | 15d | If quantitative synthesis is not appropriate, describe the type of summary planned - NA | |
| Meta-bias(es) | | | 16 | Specify any planned assessment of meta-bias(es) (such as publication bias across studies, selective reporting within studies) – page 13 | |
| Confidence in cumulative evidence | | | 17 | Describe how the strength of the body of evidence will be assessed (such as GRADE)  – page 14 | |
| *** It is strongly recommended that this checklist be read in conjunction with the PRISMA-P Explanation and Elaboration (cite when available) for important clarification on the items. Amendments to a review protocol should be tracked and dated. The copyright for PRISMA-P (including checklist) is held by the PRISMA-P Group and is distributed under a Creative Commons Attribution Licence 4.0.**  *From: Shamseer L, Moher D, Clarke M, Ghersi D, Liberati A, Petticrew M, Shekelle P, Stewart L, PRISMA-P Group. Preferred reporting items for systematic review and meta-analysis protocols (PRISMA-P) 2015: elaboration and explanation. BMJ. 2015 Jan 2;349(jan02 1):g7647.* | | | | | |

**SUPPLEMENT B**

**The PubMed search strategy including MeSH terms**

Date run: 12/20/2024 7:25:48

| ID | Search | Hits |
| --- | --- | --- |
| #1 | "quadratus lumborum block"[Title/Abstract] OR "anterior quadratus lumborum block"[Title/Abstract] OR "posterior quadratus lumborum block"[Title/Abstract] OR "lateral quadratus lumborum block"[Title/Abstract] OR "intramuscular quadratus lumborum block"[Title/Abstract] OR "transmuscular quadratus lumborum block"[Title/Abstract] OR "QLB"[Title/Abstract] | 640 |
| #2 | ("Supra-arcuate"[All Fields] AND ("ligament s"[All Fields] OR "ligaments"[MeSH Terms] OR "ligaments"[All Fields] OR "Ligament"[All Fields])) OR "lateral supra arcuate ligament"[Title/Abstract] | 11 |
| #3 | #1AND #2 | 11 |
| #4 | "QLB-LSAL"[Title/Abstract] OR "QLBLSAL"[Title/Abstract] | 5 |
| #5 | #3 OR #4 | 11 |
| #6 | "Pain, Postoperative"[Mesh] | 53,889 |
| #7 | "postsurgical pain"[Title/Abstract] OR "pain postsurgical"[Title/Abstract] OR "post surgical pain"[Title/Abstract] OR "pain post surgical"[Title/Abstract] OR "post surgical pain"[Title/Abstract] OR "post operative pain"[Title/Abstract] OR "post operative pain"[Title/Abstract] OR "post operative pains"[Title/Abstract] OR "postoperative pain"[Title/Abstract] OR "pain post operative"[Title/Abstract] OR "pain post operative"[Title/Abstract] OR "postoperative pain acute"[Title/Abstract] OR "pain acute postoperative"[Title/Abstract] OR "post operative pain acute"[Title/Abstract] OR "pain acute post operative"[Title/Abstract] OR "post operative pain acute"[Title/Abstract] OR "acute postoperative pain"[Title/Abstract] OR "acute post operative pain"[Title/Abstract] OR "acute post operative pain"[Title/Abstract] OR "postoperative pain chronic"[Title/Abstract] OR "pain chronic postoperative"[Title/Abstract] OR "chronic postsurgical pain"[Title/Abstract] OR ((("Chronic"[All Fields] OR "chronical"[All Fields] OR "chronically"[All Fields] OR "chronicities"[All Fields] OR "chronicity"[All Fields] OR "chronicization"[All Fields] OR "chronics"[All Fields]) AND ("Postsurgical"[All Fields] OR "postsurgically"[All Fields])) AND "Pains"[Title/Abstract]) OR "pain chronic postsurgical"[Title/Abstract] OR "postsurgical pain chronic"[Title/Abstract] OR "persistent postsurgical pain"[Title/Abstract] OR "pain persistent postsurgical"[Title/Abstract] OR (("Postsurgical"[All Fields] OR "postsurgically"[All Fields]) AND "pain persistent"[Title/Abstract]) OR "chronic post operative pain"[Title/Abstract] OR "chronic post operative pain"[Title/Abstract] OR "post operative pain chronic"[Title/Abstract] OR "pain chronic post operative"[Title/Abstract] OR "post operative pain chronic"[Title/Abstract] OR "chronic postoperative pain"[Title/Abstract] OR "chronic post surgical pain"[Title/Abstract] OR "chronic post surgical pain"[Title/Abstract] OR "pain chronic post surgical"[Title/Abstract] OR "post surgical pain chronic"[Title/Abstract] OR "postoperative analgesia"[Title/Abstract] OR "analgesia after"[Title/Abstract] | 50,051 |
| #8 | #6 OR #7 | 77,033 |
| #9 | ("randomized controlled trial"[Publication Type] OR "controlled clinical trial"[Publication Type] OR "randomized"[Title/Abstract] OR "placebo"[Title/Abstract] OR "clinical trials as topic"[MeSH Terms:noexp] OR "randomly"[Title/Abstract] OR "trial"[Title]) NOT ("animals"[MeSH Terms] NOT ("humans"[MeSH Terms] AND "animals"[MeSH Terms])) | 1,534,225 |
| #10 | #5 AND #8AND #9 | 7 |
| ((("quadratus lumborum block"[Title/Abstract] OR "anterior quadratus lumborum block"[Title/Abstract] OR "posterior quadratus lumborum block"[Title/Abstract] OR "lateral quadratus lumborum block"[Title/Abstract] OR "intramuscular quadratus lumborum block"[Title/Abstract] OR "transmuscular quadratus lumborum block"[Title/Abstract] OR "QLB"[Title/Abstract]) AND (("Supra-arcuate"[All Fields] AND ("ligament s"[All Fields] OR "ligaments"[MeSH Terms] OR "ligaments"[All Fields] OR "Ligament"[All Fields])) OR "lateral supra arcuate ligament"[Title/Abstract])) OR ("QLB-LSAL"[Title/Abstract] OR "QLBLSAL"[Title/Abstract])) AND ("pain, postoperative"[MeSH Terms] OR ("postsurgical pain"[Title/Abstract] OR "pain postsurgical"[Title/Abstract] OR "post surgical pain"[Title/Abstract] OR "pain post surgical"[Title/Abstract] OR "post surgical pain"[Title/Abstract] OR "post operative pain"[Title/Abstract] OR "post operative pain"[Title/Abstract] OR "post operative pains"[Title/Abstract] OR "postoperative pain"[Title/Abstract] OR "pain post operative"[Title/Abstract] OR "pain post operative"[Title/Abstract] OR "postoperative pain acute"[Title/Abstract] OR "pain acute postoperative"[Title/Abstract] OR "post operative pain acute"[Title/Abstract] OR "pain acute post operative"[Title/Abstract] OR "post operative pain acute"[Title/Abstract] OR "acute postoperative pain"[Title/Abstract] OR "acute post operative pain"[Title/Abstract] OR "acute post operative pain"[Title/Abstract] OR "postoperative pain chronic"[Title/Abstract] OR "pain chronic postoperative"[Title/Abstract] OR "chronic postsurgical pain"[Title/Abstract] OR ((("Chronic"[All Fields] OR "chronical"[All Fields] OR "chronically"[All Fields] OR "chronicities"[All Fields] OR "chronicity"[All Fields] OR "chronicization"[All Fields] OR "chronics"[All Fields]) AND ("Postsurgical"[All Fields] OR "postsurgically"[All Fields])) AND "Pains"[Title/Abstract]) OR "pain chronic postsurgical"[Title/Abstract] OR "postsurgical pain chronic"[Title/Abstract] OR "persistent postsurgical pain"[Title/Abstract] OR "pain persistent postsurgical"[Title/Abstract] OR (("Postsurgical"[All Fields] OR "postsurgically"[All Fields]) AND "pain persistent"[Title/Abstract]) OR "chronic post operative pain"[Title/Abstract] OR "chronic post operative pain"[Title/Abstract] OR "post operative pain chronic"[Title/Abstract] OR "pain chronic post operative"[Title/Abstract] OR "post operative pain chronic"[Title/Abstract] OR "chronic postoperative pain"[Title/Abstract] OR "chronic post surgical pain"[Title/Abstract] OR "chronic post surgical pain"[Title/Abstract] OR "pain chronic post surgical"[Title/Abstract] OR "post surgical pain chronic"[Title/Abstract] OR "postoperative analgesia"[Title/Abstract] OR "analgesia after"[Title/Abstract])) AND (("randomized controlled trial"[Publication Type] OR "controlled clinical trial"[Publication Type] OR "randomized"[Title/Abstract] OR "placebo"[Title/Abstract] OR "clinical trials as topic"[MeSH Terms:noexp] OR "randomly"[Title/Abstract] OR "trial"[Title]) NOT ("animals"[MeSH Terms] NOT ("humans"[MeSH Terms] AND "animals"[MeSH Terms]))) | | |
